# Identification of Breast Cancer Subtype-Specific Biomarkers by Integrating Copy Number Alterations and Gene Expression Profiles

**DOI:** 10.3390/medicina57030261

**Published:** 2021-03-12

**Authors:** Claudia Cava, Mirko Pisati, Marco Frasca, Isabella Castiglioni

**Affiliations:** 1 Institute of Molecular Bioimaging and Physiology, National Research Council (IBFM-CNR), Via F. Cervi 93, Segrate-Milan, 20090 Milan, Italy; mirko.pisati@studenti.unimi.it; 2 Department of Computer Science, Università degli Studi di Milano, Via Celoria 18, 20133 Milano, Italy; marco.frasca@unimi.it; 3 Department of Physics “Giuseppe Occhialini”, University of Milan-Bicocca Piazza dell’Ateneo Nuovo, 20126 Milan, Italy; isabella.castiglioni@unimib.it

**Keywords:** copy number alteration, gene expression, breast cancer subtypes

## Abstract

*Background and Objectives*: Breast cancer is a heterogeneous disease categorized into four subtypes. Previous studies have shown that copy number alterations of several genes are implicated with the development and progression of many cancers. This study evaluates the effects of DNA copy number alterations on gene expression levels in different breast cancer subtypes. *Materials and Methods*: We performed a computational analysis integrating copy number alterations and gene expression profiles in 1024 breast cancer samples grouped into four molecular subtypes: luminal A, luminal B, HER2, and basal. *Results*: Our analyses identified several genes correlated in all subtypes such as *KIAA1967* and *MCPH1*. In addition, several subtype-specific genes that showed a significant correlation between copy number and gene expression profiles were detected: *SMARCB1*, *AZIN1*, *MTDH* in luminal A, *PPP2R5E*, *APEX1*, *GCN5* in luminal B, *TNFAIP1*, *PCYT2*, *DIABLO* in HER2, and *FAM175B*, *SENP5*, *SCAF1* in basal subtype. *Conclusions*: This study showed that computational analyses integrating copy number and gene expression can contribute to unveil the molecular mechanisms of cancer and identify new subtype-specific biomarkers.

## 1. Introduction

Breast cancer (BC) is a heterogeneous disease categorized into four subtypes: luminal A (LumA), luminal B (LumB), HER2-enriched (HER2), and basal-like (basal). The different BC subtypes can be distinguished based on the expression level of four significant biomarkers by immunohistochemistry: estrogen receptor (ER), progesterone receptor (PR), human epidermal growth factor receptor 2 (HER2), and Ki-67. Indeed, luminal A is characterized by ER positive and/or PR positive and Ki-67 < 14%, lumB by ER positive and/or PR positive and Ki-67 ≥ 14%, Her2-enriched by ER negative and PR negative and Her2 positive, and triple negative by ER negative and PR negative and Her2 negative [1]. The molecular classification, such as PAM-50, which considers the expression levels of mRNAs, defines the triple negative subtype with the term basal [2]. However, previous studies reported that there is an overlap of 80% between basal BC defined by molecular classification and triple negative BC defined by immunohistochemistry [3]. Different BC subtypes have different clinical outcomes, such as patient survival, prognosis, and relapse [1].

A correct and early diagnosis of BC subtypes is essential to give effective treatments for patients. Huge volumes of biological data derived by high-throughput sequencing technologies are publicly available in databases, such as The Cancer Genome Atlas (TCGA) and Gene Expression Omnibus (GEO). With the availability of these data, many methods that combined multi-omics data were developed to identify biological patterns and reveal new biological processes [4,5].

Genomic instability, including copy number alteration (CNA), characterizes many cancers, such as BC. Copy number alterations are changes in the DNA where the number of copies of a segment DNA can result in an amplification or deletion of a specific gene.

Recently, more and more studies showed that genomic aberrations are key events in the progression from normal to tumoral tissue [6,7]. In addition, to investigate the crucial role of genomic alterations and gene expression profiles in disease progression, some studies reported novel candidate genes by integrating gene expression and CNA profiles [8,9].

To date, different computational methods based on such integration detected driver genes [10,11,12]. One example is HIT’Ndrive: its aim was to identify patient-specific altered genes that when combined can change the expression levels of transcripts [13]. HIT’Ndrive was applied to 2200 tumors in a pan-cancer study and detected some important driver genes [13]. A probabilistic model is used by another approach to investigate how mutated genes can influence the expression of other genes [14]. Similarly, Suo et al. defined driver genes as those genes that showed a mutation and interacted with a high number of differentially expressed genes in a gene network [15]. However, only some of the current methods can discover driver genes, as many of them are optimized to identify de-regulated biological modules. In addition, none of them to our knowledge is applied to the BC molecular subtypes. Indeed, the novelty of our study is the integrative analysis of copy number alteration and gene expression profiles to identify genes commonly modulated in all BC subtypes and subtype-specific genes.

CNAs can promote tumor development via the changes of gene expression levels. However, the effects of CNAs on gene expression levels are difficult to study, as gene expression profiling is often performed on biopsies containing tumor and normal cells. Therefore, gene expression levels, which are the average expression of all cell types, are often overshadowed by the influences of non-tumor cells [16,17]. A solution is the use of genomic and gene expression profiles derived from the same patient. Previous studies reported that the integration of mutation and gene expression profiles from the same patient could explain the genetic heterogeneity of cancer [18,19]. As gene expression profiles can detect crucial genes whose expression dynamically changes, for example during the cell cycle, the integration of genomic data from the same patient can give more solid results and reduce the variability of data.

In this study, we analyzed a large dataset of copy number and gene expression profiles to identify BC subtype-specific driver genes. We performed a computational analysis correlating DNA copy number alterations and gene expression profiles in BC samples from the TCGA database. We identified the genes that showed a correlation in all BC subtypes, and BC subtypes-specific genes. Furthermore, we used two other GEO datasets to identify subtype-specific genes that were altered in at least 50% of the samples in TCGA and GEO datasets. Finally, we investigated the biological role of these subtype-specific genes through pathway enrichment analysis.

## 2. Materials and Methods

The present work introduces an analysis of copy number and gene expression profiles of paired BC samples derived by the TCGA database. We performed a correlation analysis between copy number alterations and gene expression levels for each gene, and we obtained a list of correlated genes for each BC subtype. Furthermore, we selected those genes from the list that are specific for each subtype in TCGA, namely that show a correlation in only one subtype. To validate the results and to obtain a robust signature we included in the study two independent datasets from GEO. Indeed, the final list of correlated genes contained those genes that showed an alteration in at least 50% of the samples in the two GEO datasets and TCGA.

**Figure 1:**
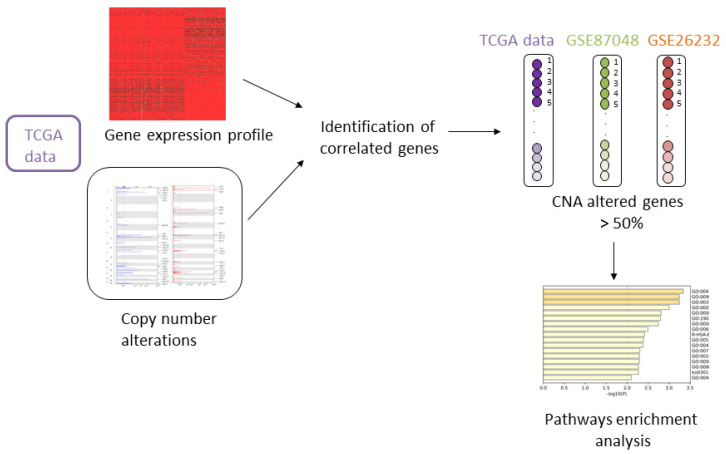
Workflow of the proposed analysis. TCGA: The Cancer Genome Atlas; CNA: Copy number alteration.

Figure 1 shows the analysis performed.

### 2.1. Data

We downloaded and pre-processed, using TCGABiolinks package [20], paired BC samples of copy number and gene expression profiles from TCGA. Copy number and gene expression data contained 548 lumA, 206 lumB, 82 HER2-positive, and 188 basal samples.

Copy number profiles of the BC samples from TCGA were downloaded using the getGistic function of the TCGABiolinks R package. The copy number matrix contained altered genes for each patient indicated with positive and negative numbers that showed amplification and deletion, respectively.

In the validation step, copy number profiles for two independent datasets from the GEO dataset (GSE87048 and GSE26232) were calculated using the package aroma.affymetrix with CRMAv2, the Circular Binary Segmentation model, and the GISTIC tool. Copy number profiles from TCGA and two GEO datasets were estimated for the whole genome. The data derived by bulk sequencing and CNAs were an average of copy numbers from thousands of cells for sample. The number of samples for each subtype is presented in Table 1. Molecular subtypes of BC samples from TCGA and GEO were established by molecular classification PAM50.

### 2.2. Integrating Copy Number and Gene Expression

We performed a Pearson correlation test between copy number alterations and gene expression levels for each gene in the TCGA data. Pearson and Spearman correlations are widely used methods to assess the influence of copy number alterations on gene expression profiles in cancer [21,22]. However, we used Pearson’s correlation because it assumes that the variables have a linear relationship and derive from a normal distribution [23]. *p*-values were adjusted with the False Discovery Rate (FDR) method. We considered two genes correlated if the correlation coefficient was >0.6 or <−0.6 and *p*-values adjusted <0.05. The correlation analysis was performed separately for each subtype.

### 2.3. Identification of Subtype-Specific CNAs

We analyzed the genes that showed a correlation between copy number alterations and gene expression levels in TCGA data, and we selected the subtype-specific genes and those common among different subtypes. For the visualization of the data, we used a Venn diagram with the R package, pheatmap.

Furthermore, we evaluated the subtype-specific genes in TCGA, and we selected those genes that were altered through copy number analysis in at least 50% of the samples in TCGA data, GSE87048, and GSE26232. We used 50% as a cut-off because it is a commonly used threshold in previous studies [24,25]. Additionally, in this step we considered the samples grouped by subtype.

### 2.4. Survival Analysis

A Kaplan–Meier plotter was used to perform a survival analysis [26]. The tool uses gene expression data and relapse free survival information of datasets downloaded from public datasets, such as GEO and TCGA.

To examine the prognostic value of a gene, the samples were divided into two groups according to median expressions of the proposed biomarkers. The differences between survival curves were estimated using the log-rank test.

We performed the analysis considering all BC samples for the common genes among BC subtypes and BC samples separately for each subtype for subtype-specific genes.

### 2.5. Pathway Analysis

We performed a pathway enrichment analysis to obtain pathways enriched with subtype-specific genes. Specifically, we used Reactome pathway enrichment analysis to obtain the enriched pathways (*p*-value < 0.01). The Reactome tool is based on a statistical test (hypergeometric distribution) that revealed Reactome pathways that are significantly enriched with subtype-specific genes [27].

## 3. Results

### 3.1. Correlation Analysis in TCGA Data

**Figure 2:**
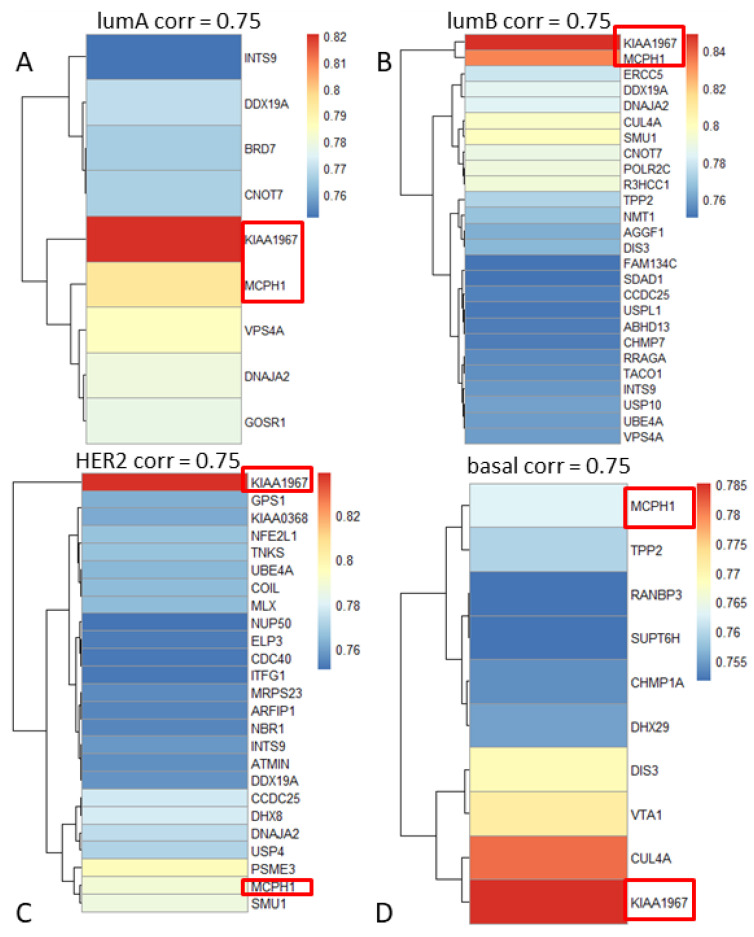
Heatmaps of correlation coefficients for each subtype: (**A**) lumA, (**B**) lumB, (**C**) HER2, and (**D**) basal (correlation coefficient ≥0.75). KIAA1967 and MCPH1 are highlighted as they showed a high correlation in all breast cancer subtypes.

Pearson correlation tests between copy number alterations and gene expression levels for each gene were performed. We found that 439 genes showed statistically significant correlations between DNA copy number and gene expression for the lumA, 970 for the lumB, 1189 for HER2, and 1029 for the basal subtype (correlation coefficient ≥0.6 or ≤−0.6, *p*-values adjusted <0.05). Figure 2 shows the heatmaps of the results of correlations.

**Figure 3:**
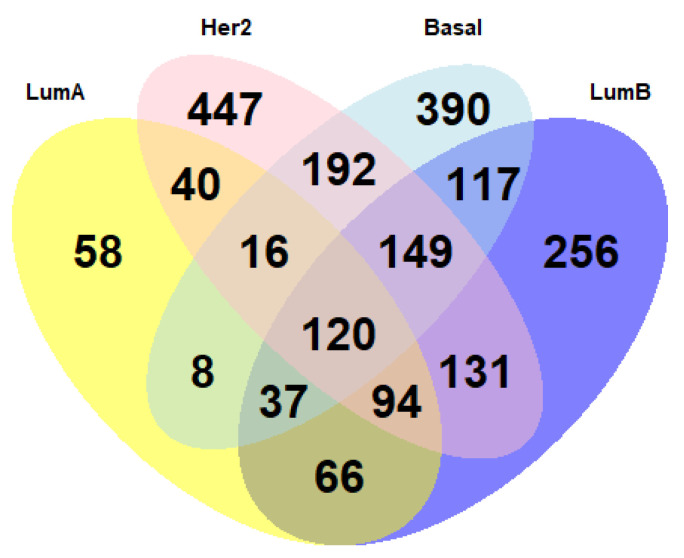
Venn diagram shows how genes whose expression is significantly correlated with the presence of copy number alterations are shared among breast cancer subtypes.

In the next steps, we focused on how the genes that showed a correlation between copy number alterations and gene expression levels in TCGA data were distributed among the various BC subtypes. Figure 3 shows with a Venn diagram the common genes among subtypes and the subtype-specific genes.

#### 3.1.1. Correlation Analysis: Common Genes among Breast Cancer Subtypes

In this step we presented the genes commonly correlated between copy number and gene expression profiles in all BC molecular subtypes.

We focused on two genes, *KIAA1967* and *MCPH1*, because, as reported in Figure 2, they obtained a high correlation coefficient in all BC subtypes. *KIAA1967*, a tumor suppressor gene, has a high correlation coefficient in all BC subtypes (0.8647 in LumA, 0.849 in LumB, 0.839 in Her2, and 0.778 in basal). Figure 3 shows the distribution of the copy number alterations of *KIAA1967* in the 4 BC subtypes. We noticed that the gene was mostly deleted. Indeed, it was deleted in 50% of LumA samples, and in 35% of the samples, there were no alterations. Only a small fraction of samples (15%) showed a gene amplification. As in lumA, also in other BC subtypes, *KIAA1967* was mostly deleted: 59% of the lumB samples, 66% of the HER2 samples, and 60% of the basal samples.

Another gene that, like *KIAA1967*, showed a high correlation value (0.795 in LumA, 0.834 in LumB, 0.789 in Her2, and 0.763 in basal) was the *MCPH1* gene. Like KIAA1967, MCPH1 was mostly deleted: 47% of the lumA samples, 56% of the lumB samples, 66% of the HER2 samples, and 59% of the basal samples.

Figure S1 shows the distribution of the copy number alterations of *KIAA1967* and *MCPH1* in the various BC subtypes.

#### 3.1.2. Correlation Analysis: Subtype-Specific Genes

We obtained 58 subtype-specific genes exclusive for the lumA. Among these, we focused on those genes that had high correlation values in the Pearson test, such as *SMARCB1* (corr = 0.67, *p*-value < 0.001), *AZIN1* (corr = 0.66, *p*-value < 0.001), and *MTDH* (corr = 0.63, *p*-value < 0.001). We performed the same procedure for lumB, HER2, and basal.

For the lumB, we obtained 256 genes including the genes with a high correlation *PPP2R5E* (corr = 0.76, *p*-value < 0.001), *APEX1* (corr = 0.7, *p*-value < 0.001), and *GCN5* (corr = 0.7, *p*-value < 0.001); for the HER2 we identified 447 genes including *TNFAIP1* (corr = 0.80, *p*-value < 0.001), *PCYT2* (corr = 0.77, *p*-value < 0.001), and *DIABLO* (corr = 0.71, *p*-value < 0.001); and for basal we obtained 390 genes such as *FAM175B* (corr = 0.76, *p*-value < 0.001), *SENP5* (corr = 0.72, *p*-value < 0.001), and *SCAF1* (corr = 0.71, *p*-value < 0.001).

### 3.2. Survival Analysis

**Figure 4:**
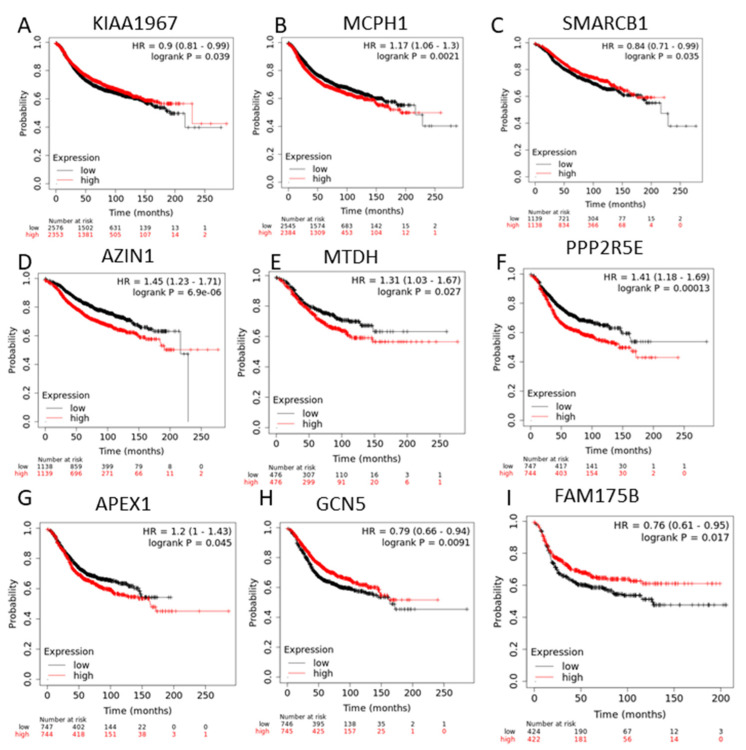
Survival analysis applied to genes whose expression is significantly correlated with the presence of copy number alterations in all breast cancer subtypes (**A**,**B**) *KIAA1967* and *MCPH1*; and subtype-specific genes (**C**–**I**) *SMARCB1* (**C**), *AZIN1* (**D**), *MTDH* (**E**) in lumA breast cancer patients, *PPP2R5E* (**F**), *APEX1* (**G**), *GCN5* (**H**) in lumBC samples, and *FAM175B* (**I**) in basal breast cancer patients.

Survival analysis was applied to genes that showed a significant correlation between copy number and gene expression profiles (Figure 4). *KIAA1967* and *MCPH1*, mostly deleted in all BC subtypes, also demonstrated a prognostic role. Indeed, the low expression of *KIAA1967* and *MCPH1* was associated with a poor prognosis.

Survival analysis was performed on *SMARCB1*, *AZIN1*, and *MTDH*, which are lumA-specific genes. Low expression of *SMARCB1* and high expression of *AZIN1*, and *MTDH* were correlated with a recurrence free survival in patients with lumA. *PPP2R5E*, *APEX1*, and *GCN5*, lumB-specific genes, were associated with a poor prognosis; high expression of *PPP2R5E* and *APEX1*, and low expression of *GCN5* showed poor recurrence free survival in patients with lumB. *FAM175B*, one of the basal-specific genes, was correlated with recurrence free survival in basal BC patients.

### 3.3. Analysis of GEO Datasets

As we obtained copy number alterations of TCGA, GSE87048, and GSE26232 samples, we focused on the subtype-specific genes that have an alteration in at least 50% of the samples for each of the 3 datasets. Although RNA-Seq used in TCGA data and microarray used in GEO datasets are different technologies, they showed a high degree of concordance. This suggests that the identification of driver genes that show a consistency of results from different technologies is an important aspect of our study.

We found that 29 out of 58 genes in lumA, 90/256 in lumB, 40/447 in HER2, and 23/390 in basal were altered in at least 50% of the samples in all three datasets. Table S1 shows the list of these genes. We obtained a good percentage of consistency in the three datasets (29/58, 50%) in lumA; 35% (90/256) of genes were reproducible in the three datasets for lumB.

### 3.4. Pathway Enrichment Analysis

We examined the biological pathways associated with subtype-specific genes. Table 2 shows the top 3 pathways enriched with the genes.

We obtained for lumA the following pathways: SUMOylation of SUMOylation proteins, SUMOylation of ubiquitinylation proteins, and defective intrinsic pathway for apoptosis due to p14ARF loss of function.

The pathways cellular senescence, oxidative stress induced senescence, and disassembly of the destruction complex and recruitment of AXIN to the membrane were identified by the analysis for the lumB.

The pathways enriched with 40 HER2-specific genes were aryl hydrocarbon receptor signaling, VxPx cargo-targeting to cilium, and amplification of signal from the kinetochores.

The pathways enriched with 23 basal-specific genes were regulation of cholesterol biosynthesis by SREBP (SREBF), activation of gene expression by SREBF (SREBP), and metabolism of steroids.

## 4. Discussion

In this study we performed an analysis pipeline integrating copy number, gene expression, and clinical data. This analysis allowed for the identification of common genes among BC subtypes and subtype-specific genes, which may be potential candidates for personalized treatment. Unlike previous approaches, our analysis does not simply produce gene drivers, but we studied the effect of DNA copy number alteration on gene expression levels in the different stages of breast cancer progression defined by molecular subtypes.

In the first step of our study, we analyzed the correlation between copy number alterations and gene expression levels for each gene and for each subtype in 1024 BC samples of TCGA data. We obtained 439 genes correlated in lumA, 970 in lumB, 1189 in HER2, and 1029 in basal subtype.

We focused on the genes with a high correlation obtained in all subtypes. Specifically, a high correlation coefficient in all subtypes was reported for two genes, *KIAA1967* and *MCPH1*.

*DBC1*/*KIAA1967* is a tumor suppressor gene that regulates p53-signaling through the inhibition of SIRT1 deacetylase. However, although *SIRT1* plays a vital role in carcinogenesis by regulating cell proliferation, survival, and death, its role in BC remains controversial [28]. *DBC1*/*KIAA1967* encodes a CCAR2 protein whose role is debated. It was reported that in squamous cell cancer, the loss of CCAR2 in mice results in cell cycle progression, suggesting that CCAR2 may function as a tumor suppressor. In addition, it was hypothesized that CCAR2 plays a role in promoting the stability of the transcription factors RFX1 and CREB1 required for proliferation [29].

However, the role of *DBC1/KIAA1967* is controversial, as in some studies it has been reported to be up-regulated and in others down-regulated within the same tumor [30]. A recent study investigated the clinical value of *DBC1/KIAA1967* in hepatocellular carcinoma, analyzing its prognostic ability with survival analysis; a higher expression of *DBC1/KIAA1967* reduced overall survival and disease free survival [30].

Survival analyses performed in our study demonstrated the prognostic role of *DBC1*/*KIAA1967* in BC; its low expression is associated with a poor prognosis.

The second gene with a high correlation in all subtype is *MCPH1*. Previous studies demonstrated that the tumor suppressor gene *MCPH1* is significantly associated with BC susceptibility in hereditary and non-hereditary BC [31]. These data therefore suggested that this gene is involved in the development of BC, despite its role needing to be further analyzed. In addition, mutated *MCPH1* down-regulates histone genes and leads to migration and invasion of the cells [32]. Frequent deletions and methylation of *MCPH1* were reported in many cancers, including BC, and were also associated with tumor stages in BC [33]. An important clinical role of *MCPH1* was demonstrated in our study; low expression of *MCPH1* is associated with a poor relapse free-survival.

Furthermore, in our study we focused on the subtype-specific genes that showed a correlation between copy number alterations and gene expression levels in TCGA data. Among 439 genes correlated in lumA, we found 58 lumA specific genes. The top three genes with the highest correlation were *SMARCB1* (corr = 0.67), *AZIN1* (corr = 0.66), and *MTDH* (corr = 0.63).

*SMARCB1* was the lumA-specific gene with the highest correlation coefficient. It encodes a member of the ATP-dependent family and plays an important role in chromatin modelling, enabling the entry of transcription factors to DNA. A recent study demonstrated that the most frequent alterations of *SMARCB1* in human cancer are the deletions [34]. However, it is not completely clear what is the role of *SMARCB1* in BC [34,35].

The second gene with a high correlation coefficient is *AZIN1*. A previous study analyzed the role of *AZIN1* in polyamine homeostasis and cell proliferation in BC cells. Polyamines are an important pathway for different cellular functions, including cell growth [36]. Their intracellular concentrations are controlled by a complex network of regulatory mechanisms, in which *AZIN1* plays a key role. *AZIN1* decreases cellular polyamine by downregulating the enzyme that catalyzes the biosynthesis of polyamine, ornithine decarboxylase (ODC), and the absorption of polyamines [36]. The activity of *AZIN1* is repressed by the binding of a protein, called Az inhibitor (AzI), which is an enzymatically inactive homolog of ODC. Two forms of AzI have been described, namely AzI1, which is omnipresent, and AzI2, which is expressed in the brain and testes. The overexpression of *AZIN1* increases cell proliferation with a simultaneous increase in ODC activity and putrescine content [36].

*MTDH*, an oncogene, has been associated with an aggressive phenotype, poor prognosis, and chemo-resistance in BC [37]. A previous study described its possible mechanism of action in cancer; HIF-1 can bind to the *MTDH* promoter and regulate *MTDH* expression [37]. Other studies reported that *MTDH* can regulate two biological pathways involved in tumorigenesis and metastasis, the NF-κB and MAPK pathways [38].

For lumB we obtained 256 out of 970 specific genes. Additionally, in this case we focused on the genes with the highest correlation, namely *PPP2R5E*, *APEX1*, and *GCN5*.

The *PPP2R5E* gene, encoding protein phosphatase 2A (PP2A), is an important cellular phosphatase and plays key regulatory roles in growth, differentiation, and apoptosis [39]. Given the wide range of cellular functions of PP2A, its activity is tightly regulated to maintain cellular homeostasis [39].

The second lumB-specific gene is *APEX1*. Genetic alterations in genes that code for proteins that play a role in DNA repair pathways and in homologous recombination of DNA such as *APEX1*, *BRCA1*, *BRCA2*, *XRCC2*, *XRCC3*, *ATM*, *CHEK2*, *PALB2*, *RAD51*, and *XPD* have been implicated in BC [40]. APEX1 is a multifunctional protein that plays a central role in the base excision repair pathway. The APEX1 gene is highly polymorphic in cancer patients and has a role in the accumulation of the apurine/apyrimidine site in DNA and consequently may lead to an increased risk of cancer development [41].

*GCN5*, also known as *KAT2A*, a prototype of histone acetyltransferase (HAT), is involved in a variety of cellular processes and regulates downstream target genes by acetylation of different lysine residues (H3K9, H3K14, H3K18, and H3K23) in the core of histone H3 [42]. Furthermore, *GCN5* modulates the function of non-histone proteins, such as transcription factors, by transferring an acetyl group to lysine residues. *GCN5* is also involved in cell cycle progression [42].

For HER2 we identified 447 genes, including *TNFAIP1*, *PCYT2*, and *DIABLO*.

A gene highly correlated and specific for the HER-2 tumor subtype is the *TNFAIP1* gene (corr = 0.8). Previous studies suggested that the *CSAGA*, *TNFAIP1*, and *POLDIP2* complex represents a gene module significantly associated with the amplification of the genomic region on 17q11.2 and correlated with the expression of ERBB2 in BC [43]. The co-expression pattern of this complex correlates with histological grades and a poor prognosis in BC when overexpressed [43].

A second gene analyzed, for the same reasons as the previous one, was the *PCYT2* gene (corr = 0.77). A previous study, based on metabolome analyses, demonstrated that glutamine deprivation leads to the accumulation of phosphoethanolamine (PEtn) in cancer cells through downregulation of cytidyltransferase PEtn (PCYT2) [44]. Accumulation of PEtn was correlated with tumor growth in nutrient-deficient conditions. *PCYT2* suppression was partially mediated by downregulation of the transcription factor ELF3. Furthermore, PCYT2 overexpression reduced PEtn levels and tumor growth [44]. *PCYT2* could represent a target in novel metabolic strategies for cancer [45].

Another gene with a high correlation was the *DIABLO* gene (corr = 0.71).

Since the Smac/DIABLO protein is involved in the mechanisms of apoptosis, it might be expected that the expression of this protein decreases with tumor development. A previous study confirmed this hypothesis, as Smac/DIABLO protein expression was significantly lower in stage 2 and stage 3 of BC than in stage 1 [46]. Furthermore, there was a weak correlation between low Smac/DIABLO protein expression and cancer embolism in minor blood and lymphatic vessels. In conclusion, the study indicated that Smac/DIABLO expression is inversely related to the tumor stage, which may suggest that this protein may play an important role in BC development [46].

For basal subtypes, we obtained 390 specific genes such as *FAM175B*, *SENP5*, and *SCAF1*.

The function of *FAM175B*, also known as *ABRO1* and *KIAA0157*, is largely unknown. Recent studies have revealed that *ABRO1* is a novel tumor suppressor by regulating the stability and functionality of p53 signaling. It plays an important role in tumor suppression and DNA damage response [47]. A recent study demonstrated that *ABRO1* overexpression stabilizes p53 and inhibits the growth of p53-expressing wild-type tumor cells, suggesting that the inhibition of cell growth by *ABRO1* upregulation is dependent on p53 status [47]. Furthermore, it was shown that *ABRO1* overexpression causes cell cycle arrest in the G1 phase, which is p53-dependent. These results suggest that *ABRO1* can suppress tumorigenesis. The role of *ABRO1* may be clinically relevant, because *ABRO1* protein levels are reduced in several cancerous tissues, including liver, kidney, breast, and thyroid cancers, and a higher *ABRO1* expression level correlates with better survival in patients [47].

A second important gene for the basal subtype is the *SENP5* gene. The downregulation of *SENP5* expression is associated with a good prognosis among BC patients. A previous study reported that the silencing of *SENP5* leads to the inhibition of growth, proliferation, and invasion in BC cell lines [48]. These changes are driven by the regulation of TGFβRI levels. One of TGFβRI target genes, *MMP9*, which plays a key role in degrading the extracellular matrix and contributes to invasion, is dramatically under-regulated by the silencing of *SENP5*. These data suggested the involvement of the SENP5-TGFβ-*MMP9* cascade in BC [48].

*SCAF1* is one of the basal-specific genes with a higher correlation coefficient between copy number alteration and gene expression levels in TCGA data. Elevated expression of *SCAF1* was observed in a previous study in 31/81 (38.3%) BC tissues and was found to be more frequent in patients with tumors of large size, as well as in patients with lymph node invasion [49].

From the validation analysis using two GEO independent datasets, we found that 29 out of 58 genes in lumA, 90/256 in lumB, 40/447 in HER2, and 23/390 genes in basal were altered in at least 50% of the samples in two GEO dataset and TCGA data. The lowest percentage of consistency for HER2 and basal can be explained by the low number of samples for the two GEO datasets. Indeed, GSE26232 contains only two HER2 samples, and genes found altered in 50% of the two samples may have affected the results. The same applied for the GSE87048 dataset, although it contained a few more samples (eight basal samples); this aspect could have influenced the obtained results.

We examined the biological pathways associated with 29 lumA-specific genes, and we obtained the following pathways: SUMOylation of SUMOylation proteins, SUMOylation of ubiquitinylation proteins, and defective intrinsic pathway for apoptosis due to p14ARF loss of function.

SUMO proteins are involved in different biological processes such as protein stability, cell growth, and apoptosis. It was previously reported that SUMO proteins are associated with advanced BC [50].

p14 plays a role in the cell cycle and apoptosis. A recent study demonstrated an association between the deletion of p14 and p53 signaling pathway disruption [51].

The pathways cellular senescence, oxidative stress induced senescence, and disassembly of the destruction complex and recruitment of AXIN to the membrane were identified by the enrichment analysis of 40 specific genes for the lumB.

40 HER2-specific genes were involved in 3 main pathways: Aryl hydrocarbon receptor signaling, VxPx cargo-targeting to cilium, and amplification of signal from the kinetochores.

The pathways enriched with 23 basal-specific genes were regulation of cholesterol biosynthesis by SREBP (SREBF), activation of gene expression by SREBF (SREBP), and metabolism of steroids. Previous studies reported that the under-expression of SREBP leads to a downregulation of several enzymes of fatty acids signaling. However, SREBP is upregulated in BC and associated with poor prognosis [52].

## 5. Conclusions

In conclusion, this study demonstrated the strong correlation between copy number alterations and gene expression levels of several known tumor suppressors and oncogenes. Thus, we revealed that integration analysis is crucial in discovery of therapeutic target genes in BC cancer subtypes.

## Figures and Tables

**Table 1:** Number of samples for each breast cancer subtype from The Cancer Genome Atlas (TCGA) and from two Gene Expression Omnibus (GEO) datasets.

Molecular Subtype	TCGA	GSE87048	GSE26232
**LumA**	548	37	12
**LumB**	206	20	6
**Her2**	82	12	2
**Basal**	188	8	17
**Total**	1024	77	37

**Table 2:** Pathways enriched by subtype-specific genes using Reactome.

	Pathway	*p*-Value
**29 Luminal A-Specific Genes**		
	SUMOylation of SUMOylation proteins	0.003
	SUMOylation of ubiquitinylation proteins	0.004
	Defective intrinsic pathway for apoptosis due to p14ARF loss of function	0.004
**90 Luminal B-Specific Genes**		
	Cellular senescence	0.005
	Oxidative stress induced senescence	0.001
	Disassembly of the destruction complex and recruitment of AXIN to the membrane	0.001
**40 HER2-Specific Genes**		
	Aryl hydrocarbon receptor signaling	0.0003
	VxPx cargo-targeting to cilium	0.003
	Amplification of signal from the kinetochores	0.004
**23 Basal-Specific Genes**		
	Regulation of cholesterol biosynthesis by SREBP (SREBF)	0.0002
	Activation of gene expression by SREBF (SREBP)	0.004
	Metabolism of steroids	0.004

## Data Availability

Publicly available datasets were analyzed in this study. This data can be found here: https://portal.gdc.cancer.gov/ (accessed on 13 November 2020); https://www.ncbi.nlm.nih.gov/geo/query/acc.cgi?acc=GSE87048 (accessed on 20 November 2020); https://www.ncbi.nlm.nih.gov/geo/query/acc.cgi?acc=GSE26232 (accessed on 30 November 2020).

## Supplementary Materials

The following are available online at https://www.mdpi.com/1648-9144/57/3/261/s1, Figure S1: Histograms showing the copy number distribution of the KIAA1967 and MCPH1 gene in all subtypes LumA, LumB, Her2, and Basal, Table S1: List of genes altered in at least 50% of samples in all three datasets for lumA, lumB, HER2, and basal.
