# Supplementary figures and images for: Identification of Breast Cancer Subtype-Specific Biomarkers by Integrating Copy Number Alterations and Gene Expression Profiles
Source: Medicina (Kaunas). 2021 Mar 12;57(3):261. doi: 10.3390/medicina57030261 (PMC7998437; doi:10.3390/medicina57030261)

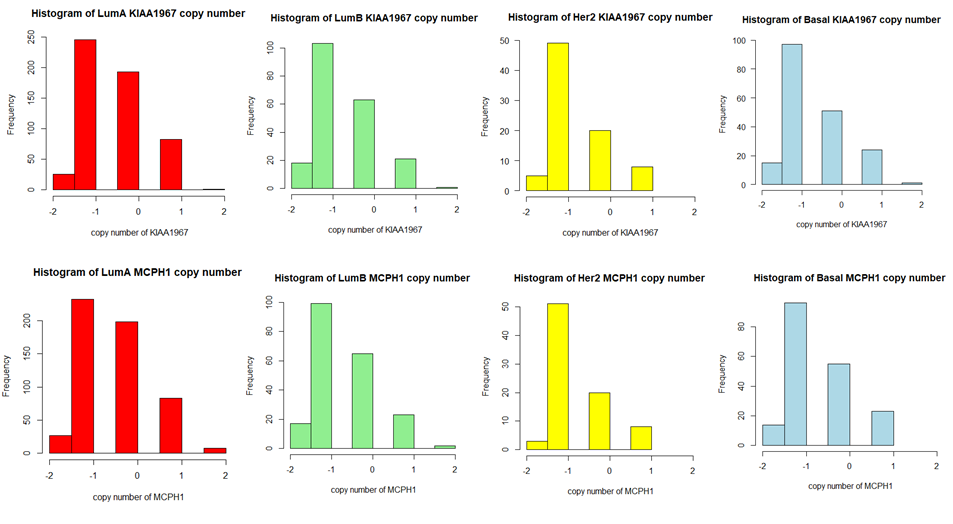

Supplement: Supplementary file 1 [file medicina-57-00261-s001.zip › supplementary/Figure S1.tif]
